# Unraveling Interdiffusion Phenomena and the Role of Nanoscale Diffusion Barriers in the Copper–Gold System

**DOI:** 10.1021/acsnano.4c08502

**Published:** 2024-10-16

**Authors:** Lilian M. Vogl, Peter Schweizer, Xavier Maeder, Ivo Utke, Andrew M. Minor, Johann Michler

**Affiliations:** † Swiss Federal Laboratories for Materials Science and Technology (Empa), 3603 Thun, Switzerland; ‡ Department of Materials Science and Engineering, University of California, Berkeley, California 94720, United States; § National Center for Electron Microscopy (NCEM), Molecular Foundry, Lawrence Berkeley National Laboratory, Berkeley, California 94720, United States; ∥ Swiss Federal Institute of Technology Lausanne (EPFL), 1015 Lausanne, Switzerland

**Keywords:** transmission electron microscopy, diffusion, in situ heating, nanowires, metals, alloys

## Abstract

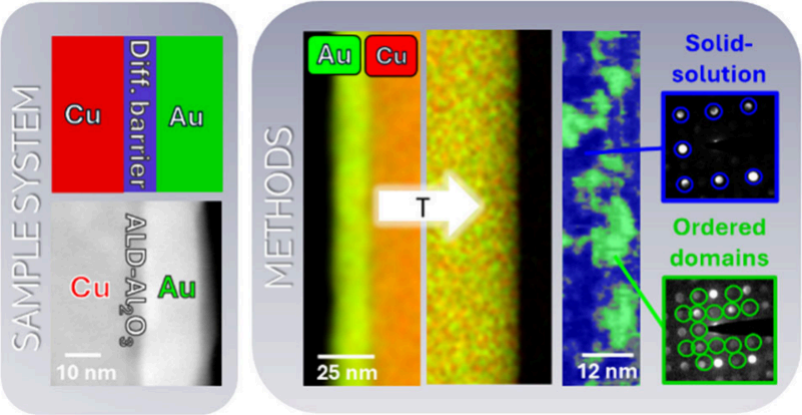

Diffusion is one of the most fundamental concepts in materials science, playing a pivotal role in materials synthesis, forming, and degradation. Of particular importance is solid state interdiffusion of metals which defines the usable parameter space for material combinations in the form of alloys. This parameter space can be explored on the macroscopic scale by using diffusion couples. However, this method reaches its limit when going to low temperatures, small scales, and when testing ultrathin diffusion barriers. Therefore, this work transfers the principle of the diffusion couples to small scales by using core–shell nanowires and *in situ* heating. This allows us to delve into the interdiffusion dynamics of copper and gold, revealing the interplay between diffusion and the disorder–order phase transition. Our *in situ* TEM experiments in combination with chemical mapping reveal the interdiffusion coefficients of Cu and Au at low temperatures and highlight the impact of ordering processes on the diffusion behavior. The formation of ordered domains within the solid-solution is examined using high-resolution imaging and nanodiffraction including strain mapping. In addition, we examine the effectiveness of ultrathin Al_2_O_3_ barrier layers to control interdiffusion of the diffusion couple. Our findings indicate that a 5 nm thick layer serves as an efficient diffusion barrier. This research provides valuable insights into the interdiffusion behavior of Cu and Au on the nanoscale, offering potential applications in the development of miniaturized integrated circuits and nanodevices.

In the field of materials science, diffusion principles are ubiquitous. However, it took a while until it was discovered that diffusion occurs not only in liquid environments but also in solids.^1^ In solid materials, diffusion generally describes a material transport by atomic motion, indicating that two adjacent metals tend to diffuse across the interface to balance their concentration profiles.^2^ This form of diffusion is utilized to synthesize functional structures,^3^ to manipulate the properties,^4^ and is directly used in engineering applications.^5,6^ However, diffusion is not always desired and can also be a critical failure of electrical devices.^7,8^ In both cases, whether intentionally inducing diffusion or suppressing the interdiffusion of two adjacent materials, a detailed understanding of the process is required. While solid-state diffusion has been researched for a long time^9,10^ and is a topic of interest,^11−13^ there are still many unknowns, especially when it comes to low temperature diffusion, the effect of diffusion barriers and the impact of the formation of phases during diffusion. The most common way of measuring solid-state diffusion is the use of diffusion couples, which is a combination of two or more pure elements with a sharp interface between them in the initial state. Upon aging, the materials start to interdiffuse, which can be tracked by various methods.^14−17^ A system that has been widely studied using diffusion couples is the Cu–Au system. This system shows complete miscibility at all compositions, along with the formation of three ordered phases (Cu_3_Au, CuAu, CuAu_3_).^18,19^ Beside the use as a catalyst material,^20,21^ the alloy is especially used in microelectronics as a metallic interconnect.^22−24^ Considering the interdiffusion of such contacts at interfaces is pivotal for designing nanoscale integrated circuits. However, conventional diffusion experiments require extended annealing times to achieve the necessary phase thickness for structural characterization.^25^ Reducing the specimen size to a minimum not only reduces the annealing time but also enables the observation of interdiffusion at the interface on a microscopic scale, even at low temperatures. Previously, the alloying dynamics of core–shell nanoparticles^26−31^ has been studied, demonstrating the powerful tool of electron microscopy imaging techniques. Building on this approach, using extended nanowires promises valuable insights into interdiffusion dynamics at order–disorder transition temperatures and with diffusion barriers.

In this work, we use Cu nanowires (NWs) coated with a Au shell as a small scale diffusion couple to enable testing of interdiffusion at small scales. *In situ* Transmission Electron Microscopy (TEM) heating experiments are performed to determine the interdiffusion coefficients of both metals by transforming the initial core–shell nanostructures into homogeneous binary NWs. We perform two different types of experiments to analyze (i) the effect of ordering and the formation of intermetallic domains within the solid-solution and (ii) the influence of diffusion barriers on the interdiffusion. The combination of techniques such as *in situ* heating, (Energy dispersive X-ray) EDX mapping, high-resolution imaging and 4D Scanning Transmission Electron Microscopy (4DSTEM) analysis including strain mapping allows us to take a detailed look into the diffusion couple on small scale. This enables us to answer questions such as how the ordering process initiates, the heterogeneity of the interdiffusion, and what constitutes an efficient diffusion barrier.

## Results

### Creation and Testing of Small Scale Diffusion Couples

**Figure 1:**
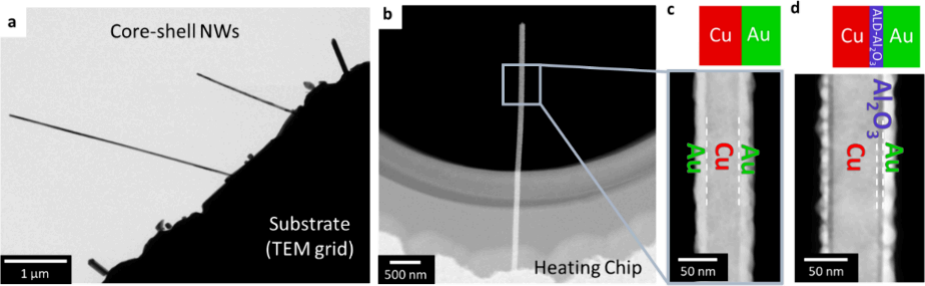
(a) Example TEM image of core–shell (Cu/Au) NWs emerging from a TEM grid. (b) STEM image of a transferred NW onto a through-hole heating chip. (c) Magnified STEM image of the NW representing a small scale diffusion couple. (d) STEM image of a diffusion couple with ALD barrier layer.

To characterize the interdiffusion of the two metals Cu and Au *in situ* in TEM, we use core–shell NWs. In an earlier publication, we established a physical vapor deposition (PVD)-based routine to produced Cu NWs (diameters between 50–100 nm), emerging from a substrate.^32^ The NWs act as core material and are then coated with a Au layer in an additional deposition step. The Au deposition (nominal thickness of 20 nm, 0.4 nm/min) is done in a Mantis Deposition Ltd., QPrep500, UK with a maximum sample rotation of 30 rpm, which ensures a consistent shell of 10–20 nm around straight NWs. Figure 1a shows an exemplary TEM image of core–shell NWs emerging from a TEM grid. For the *in situ* heating experiment, we transferred^33,34^ suitable core–shell NWs onto through-hole heating chips for a DENSsolution Wildfire TEM holder, NL, see Figure 1b. The sample preparation process is schematically illustrated in Supporting Information 1. A STEM image of a transferred NW that now serves as a small scale diffusion couple is displayed in Figure 1c. To induce a diffusion barrier layer between the two metals, the Cu NWs are coated using Atomic Layer Deposition (ALD).^35^ The process to deposit ALD-Al_2_O_3_ is a well-established routine based on the two precursors water and trimethylaluminum.^36,37^ In the past, attempts been made to use 2D materials such as graphene as an ultrathin diffusion barrier, however, factors like the adhesive behavior and defects make the application challenging.^38^ Instead, using ALD to deposit diffusion barriers offers the advantage of having precise thickness control at the angstrom scale, along with conformal growth that provides a high step coverage on a wide range of substrates.^39^ The ALD deposition was done at 120 °C in the same instrument as the PVD process for the NW growth. The combined PVD-ALD setup allowed us to directly transfer the samples between the chambers without breaking the vacuum. After preparing Cu NWs with 5 and 1 nm thick layers of ALD-Al_2_O_3_, the samples are coated with the Au layer, creating a diffusion couple separated by a barrier layer. Figure 1d shows an example STEM image of such a NW which is used as a diffusion couple with a diffusion barrier layer. To acquire the EDX mappings, *in situ* heating was paused at defined time intervals. During heating the electron beam was switched off. Additional wires on the same heating chip acted as a reference for the experiment without any electron beam exposure (Supporting Information 2). Complementary to our study, we additionally heat-treated samples under an argon flow in an external furnace equipped with a backing pump (see Supporting Information 3).

We performed different types of heating experiments to (i) analyze the effect of the ordering process and (ii) analyze the impact of diffusion barriers on the interdiffusion. The first part of the Results section presents the heating experiments done at 350 and 400 °C which are used to characterize the ordering process and the resulting transition from solid-solution to a intermetallic phase. For the Cu–Au material system (space group of pure elements: *Fm*3̅*m*) three ordered phases can be formed. Both the gold-rich CuAu_3_ phase and the copper-rich Cu_3_Au phase have an L1_2_ crystal structure (space group: *Pm*3̅*m*), and the CuAu phase shows a tetragonal structure. The initial composition of our NWs allows us to study the formation of a binary solid-solution (hereinafter called Cu_3_Au (ss)) as well as the transition to the intermetallic Cu_3_Au structure (hereinafter called Cu_3_Au (ordered)). After that, experiments to test the effect of ALD barrier layers on the diffusion couple will be presented.

### Effect of Ordering on the Interdiffusion

**Figure 2:**
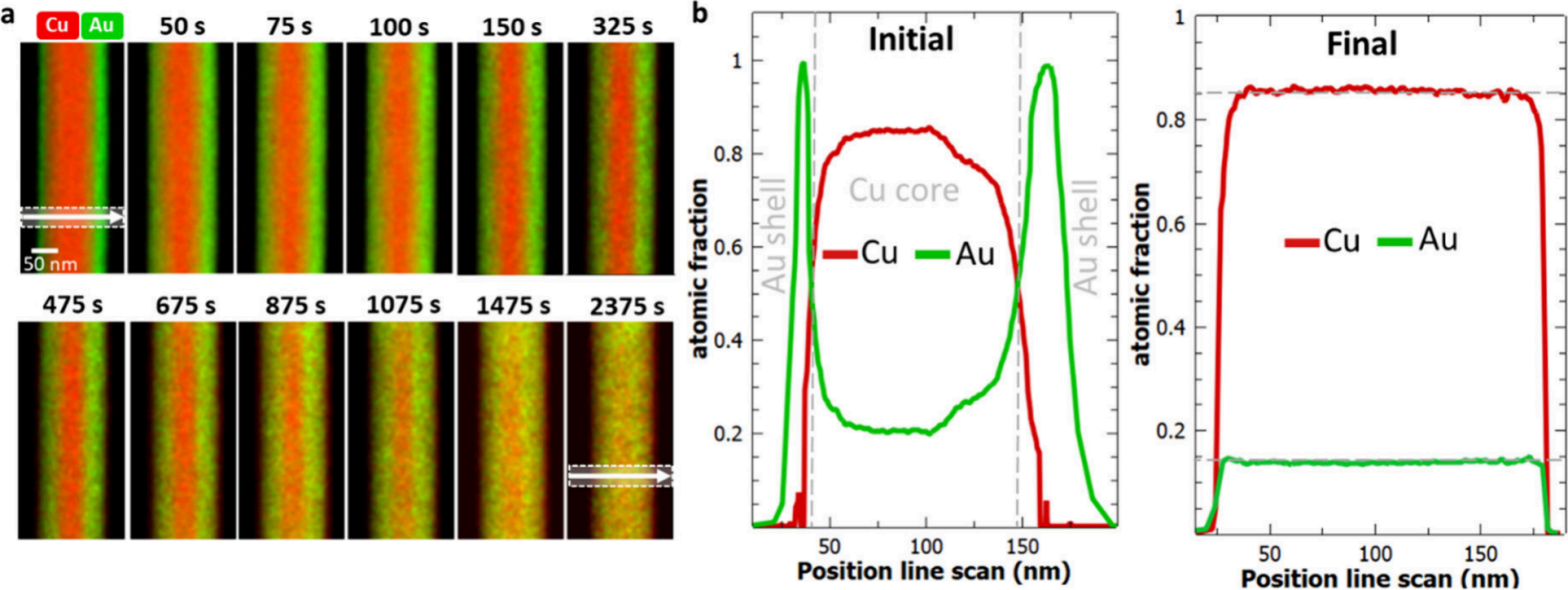
Interdiffusion at 400 °C. (a) EDX mapping (Cu, Au signal) at different time intervals acquired. (b) Comparison of line profiles before and after heating across the NW (indicated in a). After being heated, the NW has a homogeneous composition of 15% Au and 85% Cu.

Figure 2a shows EDX mappings with the proceeding time (initial-2400 s) of a core–shell NW annealed at 400 °C. The line scans before and after annealing are compared in Figure 2b. The initial NW shows a distinct Au shell around the Cu core. After heat treatment, the core–shell structure is no longer present leading to a completely intermixed Cu_3_Au (ss) NW. With proceeding time, the Au and Cu amount levels itself leading to consistent 15 atom % Au and 85 atom % Cu across the NW. This final concentration is lower than what would be expected if the gold shell was perfectly uniform. The deposition process can lead to slightly more gold being deposited on one side compared to the other, as visible in Figure 2a. For the calculation of interdiffusion coefficients we used both sides independently. Therefore, the initial thickness of the Au layer does not affect the obtained interdiffusion coefficients. Based on our experimental data, we calculated the interdiffusion coefficient using the established Wagner equation for binary alloys,^40^ taking into account the initial core–shell structure (see Supporting Information 4). We obtain constant interdiffusion coefficients of D_Cu_ = (8.8 ± 1.1) × 10^–18^ m^2^/s and D_Au_ = (3.3 ± 1.4) × 10^–18^ m^2^/s over the complete heating time. These values are close to bulk values for a random solid-solution (the equilibrium phase at this temperature and composition), as will be discussed further below.

**Figure 3:**
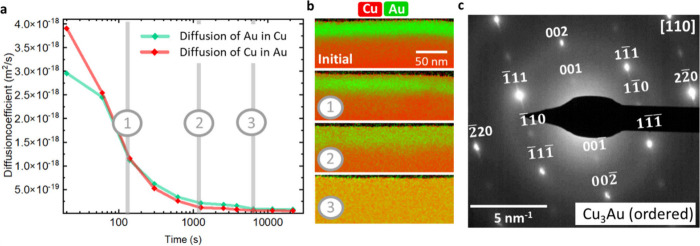
Interdiffusion at 350 °C. (a) Change of interdiffusion coefficient with proceeding heating time. After 5400 s, a constant value is reached. (b) Selected EDX mappings (Cu and Au signal) acquired after different time intervals. (c) Diffraction pattern in [110] zone axis of the NW after heating corresponding to the Cu_3_Au (ordered) phase. The position of the SAED aperture is indicated in the corresponding CTEM image in Supporting Information 5.

In the next step, we perform the same experiment at 350 °C. In contrast to the previous sample, where we achieved a complete intermixing after 2400 s at 400 °C, heating at lower temperatures requires 54000 s (factor of 22.5, total time 15 h) to achieve the same result. The increasing annealing time already implies a slowed interdiffusion process, which can be explained by the lower temperature but also by the effect of ordering. Interestingly, the calculated interdiffusion coefficients for this experiment are not constant over the complete heating time. Rather we observe a decreasing coefficient converging after 1800 s toward constant values of D_Cu_ = (8.8 ± 1.6) × 10^–20^ m^2^/s and D_Au_ = (5.5 ± 1.7) × 10^–20^ m^2^/s. Figure 3a shows the interdiffusion coefficients based on EDX mappings, which are displayed exemplarily in Figure 3b. The diffraction pattern after the heat treatment shows superlattice reflections (Figure 3c), indicating an intermetallic phase formation of Cu_3_Au (ordered) and ordering domains.

**Figure 4:**
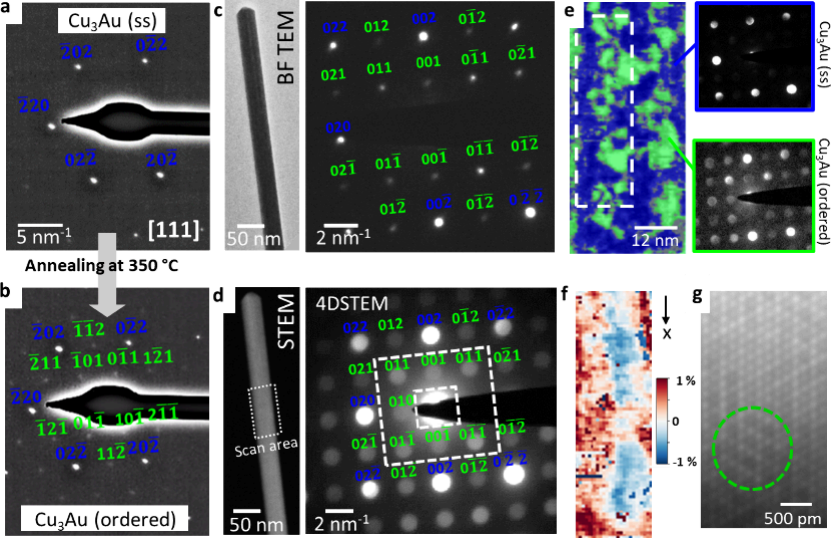
(a) Diffraction pattern in the [111] zone axis of a solid-solution NW, achieved after heating a core–shell NW at 400 °C. 220-type reflections (in blue) with *d*-spacing correspond to Cu_3_Au (ss). (b) Diffraction pattern after subsequent annealing at 350 °C. During heating, the superlattice reflections (in green) appear, indicating the formation of the ordered Cu_3_Au phase. See also the corresponding in situ video in Supporting Information 6. (c) Bright-field TEM image of a NW and the corresponding diffraction pattern in the [100] zone axis corresponding to the Cu_3_Au (ordered) phase. (d) STEM image of the same NW with marked scan area of the 4DSTEM and corresponding mean nanobeam diffraction pattern (blue: 200/220-type reflections, green: superlattice reflections). (e) Colored virtual dark field image. Green: ordered domains, showing superlattice reflections. Cu_3_Au (ordered) phase. Blue: solid-solution; absence of superlattice reflections in the nanobeam diffraction pattern. Cu_3_Au (ss) phase. The position of the virtual aperture is indicated in (d). Dashed rectangle: area of the strain map shown in (f). (g) HRSTEM image of the NW, showing regions of ordering (within the green dashed line).

After heating at 400 °C (see Figure 2), the initial core–shell structure is no longer present, and a solid solution Cu_3_Au (ss) NW is formed. Figure 4a shows the corresponding diffraction pattern with the 220-type fundamental reflections. To transform the solid-solution into an ordered lattice, we annealed the NW in an additional step at 350 °C. During heat treatment, we observe in diffraction mode the appearance of superlattice reflections (see Supporting Information 6 for the *in situ* diffraction experiment). Figure 4b shows the corresponding final diffraction pattern after annealing corresponding to the Cu_3_Au (ordered) phase. The 112-type and 110-type reflections (in green) appear due to the formation of the intermetallic Cu_3_Au phase. The transformation solid-solution/intermetallic phase is reversible, and by increasing the temperature the initial Cu_3_Au (ss) related diffraction pattern can be reobtained. Figure 4c shows the bright field image of another NW after heat treatment at 350 °C and the corresponding diffraction pattern in the [100] zone axis. The diffraction pattern of the Cu_3_Au (ordered) phase clearly shows the superlattice reflections; however, we do not get information on the ordering degree or domain formation. Therefore, we acquired a 4DSTEM map, which gives us a locally resolved diffraction pattern. The corresponding STEM image with the marked scan area is displayed in Figure 4d. The mean nanobeam diffraction pattern is equivalent to the conventional diffraction pattern, but the 4DSTEM data include local information on all scan positions. We used a rectangular shaped aperture to exclude the 220-type reflections for virtual dark field imaging. Figure 4f shows the color-coded overlay of the virtual image. The green area indicates the presence of ordering, showing in the diffraction pattern superlattice reflections. The diffraction patterns within the blue area show only 220-type reflections, which indicates a predominantly randomly arranged lattice of Cu/Au atoms. The ordered domains have sizes in the range 4–9 nm. We also evaluated the strain (see strain map in Figure 4f). The reference for the strain map is taken by averaging all patterns from the NW. Domains of negative and positive strain can be interpreted as domains where the lattice parameter slightly deviates from the average. The domains with a negative strain roughly line up with ordered areas, whereas a positive strain can be associated with solid-solution areas. In high-resolution the ordering domains are visible (see the HRSTEM image in Figure 4g). Interestingly, it seems that the ordering is more present near the edge of the NW and the disorder in the core region is higher, which might be due to the initial core–shell structure.

### Effect of Diffusion Barriers on the Interdiffusion

**Figure 5:**
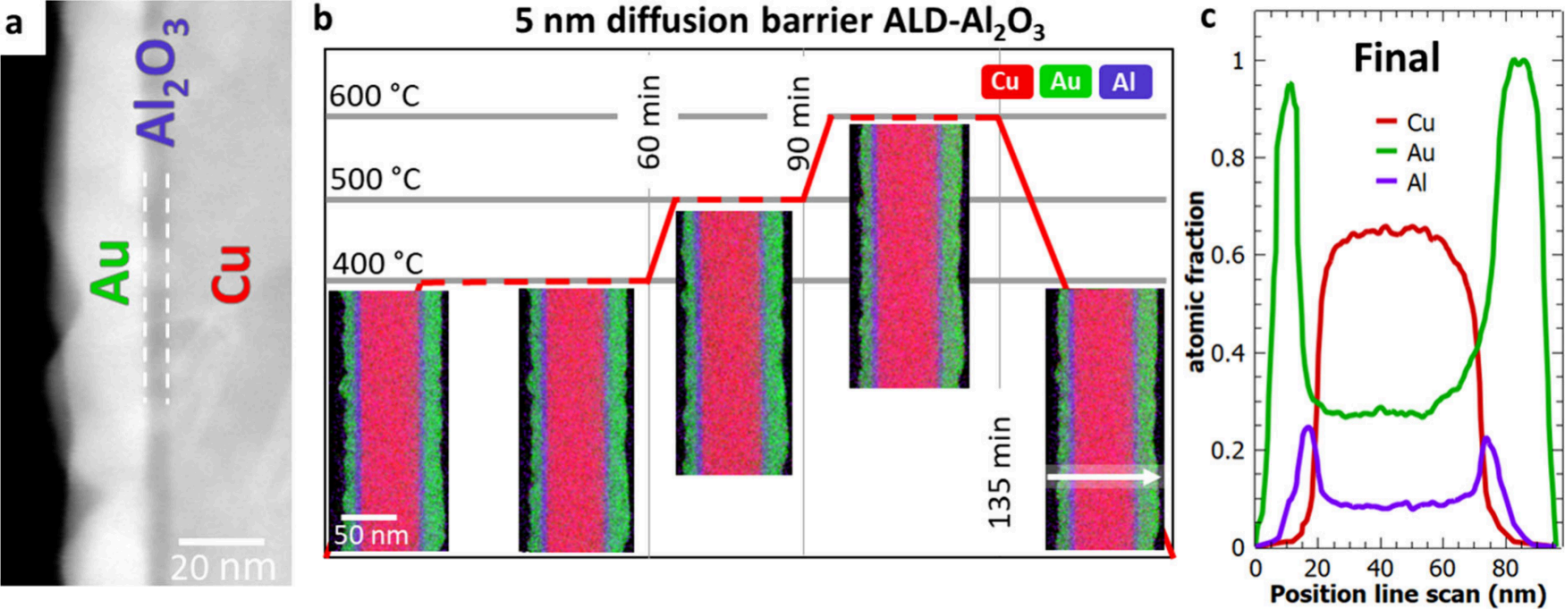
(a) Example STEM image of a core–shell NW with diffusion barrier (ALD-Al_2_O_3_). (b) Overview of the TEM heating experiment of the diffusion couple with an intermediate layer of 5 nm ALD-Al_2_O_3_. EDX mappings (Cu, Au, and Al signal) at different time intervals. (c) Line scan across the NW after heating. The position of the final line scans is shown in part (b).

In the previously described experiments, we analyzed the interdiffusion of Cu and Au at two different temperatures. To see the effect of a barrier layer on the interdiffusion process, we adapted the core–shell structure by bringing in an intermediate layer of ALD-Al_2_O_3_ between our diffusion couple. Figure 5a shows an exemplary STEM image of a sample with a diffusion barrier. Before coating the Cu NWs with a gold layer, an ALD deposition of Al_2_O_3_ was performed. We compare the effect of 5 nm (Cu-5 nm Al_2_O_3_–Au) and 1 nm (Cu-1 nm Al_2_O_3_–Au) thick ALD-Al_2_O_3_ on the interdiffusion of Cu and Au. Figure 5b gives an overview of the heating experiment performed on the 5 nm diffusion barrier (Cu-5 nm Al_2_O_3_–Au), including EDX mappings and the heating profile starting at 400 °C. Without a diffusion barrier, we observe a complete intermixing of the two metals after 40 min. We heated the Cu-5 nm Al_2_O_3_–Au sample for 60 min, but the EDX profile did not show any compositional changes in the multilayered structure. Therefore, we increased the temperature to 500 °C and acquired intermediate EDX maps for 30 min. However, no changes were observed in this case either. We increased the temperature further to 600 °C, but still no interdiffusion occurs even after 45 min of continuous heating at that temperature. Figure 5c shows the final line scan across the NW. The surface of the Au shell is slightly smoother. This indicates that the heat induces some structural changes within the surface of the shell but no interdiffusion through the diffusion barrier has occurred during the applied heating profile.

**Figure 6:**
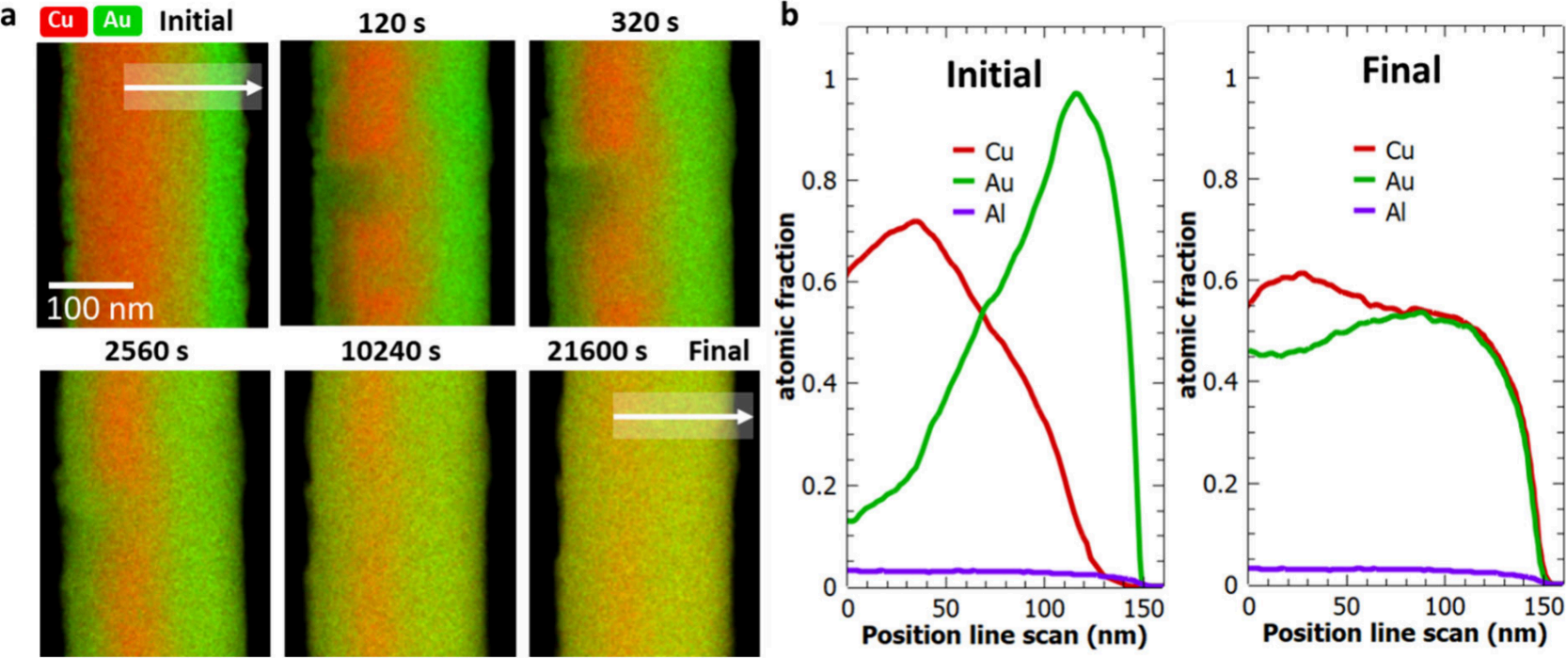
(a) Heating experiment at 400 °C of the Cu-1 nm Al_2_O_3_–Au sample. EDX mappings (Cu and Au signals) were acquired at different time intervals. The white arrow indicates the line scan position across the interface. White dashed square: weak point of the barrier layer. (b) Comparison of the initial and final line profile (Au, Cu, Al signal).

Reducing the thickness of the ALD layer changed this behavior. The Cu-1 nm Al_2_O_3_–Au sample shows interdiffusion at 400 °C, however, it is much slower compared to the pure diffusion couple. Figure 6a shows the sequence of EDX mappings for the heating experiment. Figure 6b compares the initial and final line scans across the interface. After 320 s, we observe an inhomogeneous interdiffusion of the Cu and Au through the diffusion barrier. Even after heating for 21 600 s, the copper core is still visible. During heat treatment, the metals diffuse through weak points (highlighted in Figure 5a) of the intermediate ALD layer, inducing an inhomogeneous diffusion profile. Hence, the 1 nm diffusion layer does not completely suppress the interdiffusion but significantly slowed down the process. We calculated the interdiffusion coefficient as D_Cu_ = (6.2 ± 1.1) × 10^–19^ m^2^/s and D_Au_ = (3.1 ± 1.4) × 10^–19^ m^2^/s, which is 10× slower compared to the values obtained for the pure diffusion couples.

## Discussion

### Small-Scale Diffusion Couple

The analysis of diffusion couples to generate phase diagrams and diffusion coefficients is well established. At low temperatures, determining diffusion coefficients through diffusion couples is, however, challenging due to the extended annealing times required to achieve sufficiently large diffusion profiles for analysis. This is one reason that reported interdiffusion coefficients for Cu and Au vary greatly in the temperature range of 300–400 °C covering a broad range between 10^–16^ and 10^–18^ m^2^/s.^41−43^ Moreover, the interdiffusion coefficient is also dependent on the composition. Ravi et al. analyzed the interdiffusion of Cu–Au thin films in dependence of the composition and showed a maximum interdiffusion coefficient at around 40–50 at. % Cu.^44^ For the material system Au–Ag the changing mobility is explained by the number of vacancies within the composition and the corresponding energy required for vacancy-hopping.^30^ For our study, we are in the copper-rich regime and therefore expect that the interdiffusion is dominated by the mobility of the Cu atoms and the availability of vacancies. Another aspect is that most of the data refer to the bulk. Theoretical models indicated that the phase diagram of nanostructures might be slightly different compared to the bulk counterpart, but experimental data is sparse.^37,38^ Guisbiers et al. calculated the phase diagram for Au–Cu nanoparticles indicating a shift of the congruent melting point to lower temperatures and toward higher Cu composition compared to the bulk.^47^ This trend has also been confirmed by other studies using different calculation approaches supported by key experiments.^45,48−50^ Also it is expected that for binary systems the stability regions of the intermetallic phases are reduced to lower temperatures.^51^

For the experiment at 400 °C we extracted the interdiffusion coefficient based on the EDX line profiles for Cu diffusing into Au ((8.8 ± 1.1) × 10^–18^ m^2^/s) and vice versa ((3.3 ± 1.4) × 10^–18^ m^2^/s). After heat treatment, a composition of 15 at. % Au and 85% at. % Cu was achieved. A diffraction pattern acquired after heating shows a solid-solution lattice (Figure 4a) and no intermetallic phase has been formed, which is in accordance with the phase diagram. The calculated interdiffusion coefficients are in a reasonable range, interestingly, showing a higher diffusive mobility of Cu into Au compared to Au diffusing into Cu. Solid state diffusion is mainly driven by vacancies.^52^ The vacancy formation energy of Cu (0.9 eV) is higher than that of Au (0.67 eV).^53^ This facilitates the substitutional diffusion of Cu by a vacancy mechanism, as there are more open sites available within the Au lattice. For the material system Au–Ag, a similar behavior during alloying has been reported. A. Skorikov et al. showed that the diffusion coefficient of Au–Ag core-shell nanorods at 450 °C fit well to tabulated bulk values, with a faster diffusion of Au into Ag.^26^ This has also been confirmed by the follow-up study of M. Mychinko et al.^28^ S. W. Chee et al. had a detailed look into the diffusion behavior of Au–Pd core–shell particles and observed void formation at the interface.^31^ In our study, we do not observe the so-called interface-mediated Kirkendall effect.^31^ Instead, the morphology of the NW remains unchanged, and no notch formation caused by annihilating voids is visible.

To rule out the effect of electron illumination on interdiffusion, the beam was switched off during heating and only used for EDX mapping after quenching to room temperature within 1 s.^54^ Furthermore, samples on the same heating chip were imaged only before and after the entire heating experiment. These samples show the same contrast as the “*in situ*” NWs. One example of such a NW is shown in Supporting Information 2. Finally, we performed correlative *ex situ* heating experiments in a furnace at the same temperature and time scale. These samples (see Supporting Information 3) also show uniform interdiffusion of the two elements after the same time. This leads us to believe that the electron beam effect in our experiment is negligible.

Reducing the temperature to 350 °C, however, means that the formation of an ordered phase governs the diffusion process. The effect of the ordering process during heating becomes apparent in our presented experiment. The calculated interdiffusion coefficients decrease as time progresses while approaching constant values of D_Cu_ = (8.8 ± 1.6) × 10^–20^ m^2^/s and D_Au_ = (5.5 ± 1.7) × 10^–20^ m^2^/s. The reason for the faster diffusion within the first minutes of heating can be explained by the fact that due to the relatively low interdiffusion, the equilibrium phase in the interdiffusion zone is still the solid solution. Essentially, we are measuring interdiffusion coefficients for a solid solution at 350 °C. Ordering starts only when a compositional threshold is reached at which the equilibrium phase is the ordered phase. Order means that the two elements want to occupy different sublattices, which leads to a competition of ordering and diffusion. Therefore, ordering leads to a slower interdiffusion process compared to disordered structures.^41^ The relative values are 2 orders of magnitude lower than those at 400 °C but show the same trend, with Cu diffusing into Au slightly faster compared to Au diffusing into the core material. The so-called “ordered Cu_3_Au rule”^55^ elucidates the diffusion in intermetallic binary A_3_B alloys, where the A atoms exhibit greater mobility in comparison to the B atoms.^55^ The copper-rich Cu_3_Au L1_2_ crystal structure corresponds to the fcc lattice, where Au occupies the corners and Cu occupies the face centers of the unit cell. The vacancy-mediated diffusion of Cu atoms can proceed by using the copper sublattice^56^ keeping the order of the crystal intact. In contrast, the diffusion of Au atoms necessitates movement from the gold sublattice onto the copper sublattice, disturbing the order in the process. Therefore, the reported activation energy for diffusion of Au in the ordered crystal is higher compared to Cu.^57,58^ The postanalysis of the sample shows a distinct diffraction spot (Figure 4c), corresponding to the Cu_3_Au (ordered) phase, which confirms the formation of an ordered lattice. With a lattice parameter of 4.08 Å, that of Au is larger than that of pure Cu (3.6 Å). Using Vegard’s law, the lattice parameter for a solid-solution lattice with the composition in our experiment is 3.67 Å. Compared to the lattice parameter of Cu_3_Au (ordered) with the L1_2_ structure (3.74 Å), the difference in the lattice parameters is 2%. The 4DSTEM scan revealed local ordered domains and strain (Figure 4e and f). The apparent strain at the domain boundaries is 0, which means that there is a relative strain of about 1% compared to neighboring solid-solution/ordered domains. The apparent strain in those domains (± 1%) can be explained by the difference in the lattice parameter compared to the mean lattice parameter used as reference for the strain mapping. Equivalent to heating the core–shell NWs directly at 350 °C, solid solution NWs can be subsequently annealed to induce ordering. Both strategies lead to the same type of NWs showing superlattice reflections in the diffraction pattern induced by the presence of ordered domains.

### Diffusion Barriers

**Figure 7:**
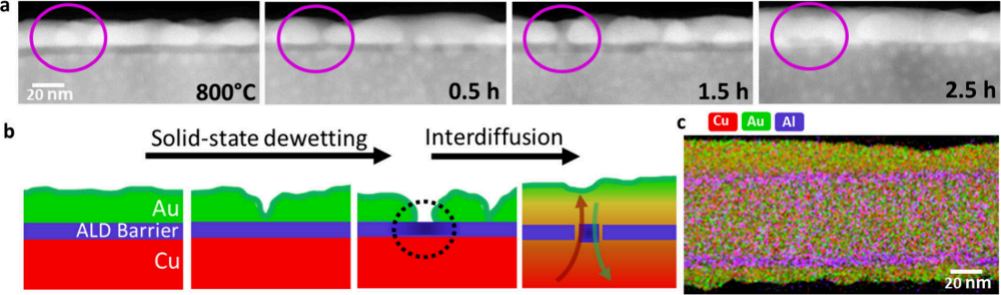
(a) STEM image sequence during heating at 800 °C. Solid-state dewetting of the Au shell induces a free surface of the ALD-barrier layer. Thermal expansion leads to cracking of ALD-Al_2_O_3_. Interdiffusion across the barrier layer. (b) Schematic illustration of the observed process. (c) EDX map (Cu, Au, Al signal) after heating at 800 °C.

We showed that already 5 nm of ALD-Al_2_O_3_ on Cu NWs can be used as an efficient diffusion barrier layer at temperatures up to 600 °C. Within that temperature range, we did not observe dewetting effects, and the multilayered nanostructures proved to be stable. To provoke an interdiffusion process, we increased the temperature further. Above 800 °C the shell shows characteristic behavior known from solid-state dewetting of thin films and we finally see an interdiffusion of Cu and Au. Figure 7a shows a STEM image sequence of the Cu-5 nm Al_2_O_3_–Au interface acquired at 800 °C. Complementary, Figure 7b illustrates the observed processes. Within the first minutes of heating, we observe solid-state dewetting of the Au shell, while the core and barrier layer do not change. With progressing dewetting, the ALD-barrier layer gets exposed to the free surface (highlighted in Figure 7b). At this weak point, cracks within the ALD-Al_2_O_3_ are induced due to the difference in thermal expansion. The thermal expansion coefficients for Cu (α_Cu_ = 17 × 10^–6^/K) and Au (α_Au_ = 14 × 10^–6^/K) are in a similar range. For ALD-Al_2_O_3_ this value depends on the deposition parameters and is in the range of α_ALD-Al_2_O_3__ = (4–8) × 10^–6^/K.^59^ The dewetting of the Au shell exposes the barrier layer, and strain within the ALD-Al_2_O_3_ will be released, which leads to defects. Further, the interdiffusion of the Cu and Au will take place through this weak point, and the barrier-layer becomes more and more permeable, which also might be caused by dewetting of the Al_2_O_3_. Figure 7c shows an EDX map after heating. The initially separated diffusion couple is now completely intermixed; however, the ALD-barrier remains at its position and did not diffuse significantly itself.

Decreasing the thickness of the ALD layer down to 1 nm weakens the barrier effect for interdiffusion. The surface quality of the Cu NW crucially affects the conformality of the ALD coating and therefore the ability to suppress the intermixing of the two metals. Metal NWs produced by advanced PVD show usually some surface inhomogeneities like voids.^34^ In contrast to 5 nm, 1 nm ALD-Al_2_O_3_ may not be thick enough to have a consistent conformal layer (see Supporting Information 7) using the standard deposition parameters in our deposition system, which are described in the Methods section. Incomplete surface coverage within the first few cycles of the ALD process^37^ induces weak points within the diffusion barrier which crucially affect the performance of the interdiffusion barrier. However, the diffusion process itself is still slowed down as the intermixing has to happen across the weak points, inducing an uneven diffusion profile. Our reference experiment shows a complete interdiffusion after 40 min of heating at 400 °C. The 1 nm barrier layer remains stable, and no additional weak points are induced during heating; therefore, the interdiffusion only occurs across positions where the initial ALD was not of sufficient quality. The Cu core was still present after 6 h of heating, meaning that the diffusion process is slowed by at least a factor of 10. This demonstrates the ability to control the interdiffusion of metals on small scales by bringing in nanometer-thick ALD layers. For a reliable diffusion barrier, we propose to use at least 5 nm of ALD-Al_2_O_3_, suppressing the entire interdiffusion of the metals and being stable up to 600 °C. Typical barrier layers/liners for copper interconnects in microelectronics are in the same thickness range (3–10 nm).^60,61^ Increasing the thickness would not improve the barrier effectiveness but rather would hinder the downscaling progress toward the fabrication of integrated circuits with limited space. We expect that other materials (e.g., nitrides) might perform even better as diffusion barriers and could be studied with our approach.

## Conclusion

Using defined small-scale model systems as diffusion couples, we have shown that one can get detailed insight into the interdiffusion and ordering processes. This specific approach can be applied to any material combination in which there is a suitable synthesis route for NWs. Compared to similar studies with nanoparticles,^26,28^ NWs have the advantage that diffusion can be tested along an extended interface which can increase the precision of interdiffusion calculations and is advantageous for testing weak points within diffusion barriers. Nevertheless, other nanostructures such as nanoparticles or rods offer other unique advantages for measuring nanoscale diffusion e.g. effect of shape. The temperature range of our methods is limited by the available heating equipment, which typically allows for temperatures between room temperature and 1200 °C. At the bulk scale, the analysis of diffusion profiles, especially at low temperatures, is prone to error, which is reflected in discrepancies in the literature values. Instead, Cu NWs coated with an Au shell allowed us to observe the interdiffusion profile *in situ* in the TEM within a reasonable experimental time frame. The combination of spectroscopy and 4DSTEM has already proven to be a powerful tool for analyzing nanostructures.^62^ Our values of the interdiffusion coefficients obtained from the EDX mappings are within a reasonable range for the Cu–Au system, showing the effect of ordering on the diffusion process. 4DSTEM shows the presence of ordered domains inducing strain on the boundaries. In addition, nanoscale diffusion barriers have been tested. We found that already 5 nm of ALD-Al_2_O_3_ is sufficient to suppress interdiffusion entirely up to 700 °C. A 1 nm layer slowed interdiffusion by a factor of 10 but still allowed for material exchange. This kind of testing of diffusion barriers is important for improving the longevity of metallic interconnects in microelectronics.^63,64^ We expect the use of small scale diffusion couples to be beneficial to a variety of different materials questions.

## Methods

The TEM analysis was performed on a Titan Themis 200 G3, Thermo Fisher Scientific, US at 200 kV at Empa Thun, Switzerland and on a ThemIS 60-300, Thermo Fisher Scientific, US at 300 kV at NCEM Berkeley, US. For the *in situ* heating experiments, samples have been transferred onto heating chips by using the gas injection system (Carbon precursor) and the integrated nanomanipulator of a Focused Ion Beam (FIB). 4DSTEM data have been acquired with a Gatan K2 Camera (dwell time 100 ms, step size 1 nm, probe size = 1.5 nm, convergence angle = 0.6 mrad). For analysis of the 4DSTEM data set (virtual dark field imaging, strain evaluation) the open source python package py4DSTEM^65^ was used. The strain mapping was performed using the whole pattern fitting method.^66^ The EDX mapping (Quantification has been performed after binning by 2) is done with a Bruker SuperX EDS detection system, US. To acquire the EDX data, the heating process has been interrupted, and the sample quenched to room temperature within 1 s. The following parameters were used for EDX mappings: 800 pA current, 1.5 nm, stepsize, 16 μs dwell time, and 200 accumulated frames. The average count in each pixel of the EDX maps was 115. The line scans were averaged over 400 pixels, leading to a total number of counts of 46000 at each data point. Quantification was performed by using the Cliff–Lorimer method using Powell ionization cross sections. After finishing the EDX maps, the beam was blanked, and the sample was brought back to the annealing temperature within 1 s. The total dose after the experiment on the nanowires was in the range of 1.2 × 10^7^ e^–^nm^–2^. Details about the sample preparation are given in Supporting Information 1.

## Supporting Information Available

*In situ* TEM heating experiment in diffraction mode. (MP4)
Schematic illustration of the sample preparation: Growth of NWs and Transfer onto heating chips; Example of NW heated under beam-off conditions; Example of *ex situ* annealed NWs in a furnace; Using core–shell NWs as small-scale diffusion couple. Calculation of diffusion coefficients: Comparison of calculated concentration profiles and experimental data; Corresponding CTEM image of the diffraction pattern shown in Figure 3c. The position of the SAED aperture is indicated with a white circle; EDX map and STEM image of a 1 nm Al_2_O_3_ shell on a Cu NW. (PDF)
