# Supplementary material for: Unraveling Interdiffusion Phenomena and the Role of Nanoscale Diffusion Barriers in the Copper–Gold System
Source: ACS Nano. 2024 Oct 16;18(43):29658–66. doi: 10.1021/acsnano.4c08502 (PMC11526422; doi:10.1021/acsnano.4c08502)
Supplement: Supplementary file 2 — nn4c08502_si_002.pdf [file nn4c08502_si_002.pdf]

Supporting Information

Unraveling Interdiffusion Phenomena and the Role of Nanoscale Diffusion Barriers in the Copper-Gold System

Lilian M. Vogl[12], Peter Schweizer[13], Xavier Maeder[1], Ivo Utke[1], Andrew M. Minor[23], Johann Michler[14]*

1 Swiss Federal Laboratories for Materials Science and Technology (Empa), 3603 Thun, Switzerland

2 Department of Materials Science and Engineering, University of California, Berkeley, CA 94720, United States of America

3 National Center for Electron Microscopy (NCEM), Molecular Foundry, Lawrence Berkeley National Laboratory, CA 94720, United States of America

4 Swiss Federal Institute of Technology Lausanne (EPFL), 1015 Lausanne, Switzerland

*Corresponding author: Lilian. M. Vogl

Supporting Information 1

Schematic illustration of the sample preparation process. For more details, the reader is referred to the following publications. Growth of NWs[1–3], NW transfer[4,5]. The nanowire synthesis was done in a custom-built PVD-ALD system, which allows to directly transfer samples between the chambers without breaking the vacuum. For the study, samples have been heated *in situ* within the TEM. For comparison, samples also have been heated ex situ in a furnace in argon atmosphere. For the *ex situ* experiment, the NWs have been mechanically transferred on conventional TEM grids with carbon film (gently sliding the TEM grid over the sample surface with freestanding NWs).

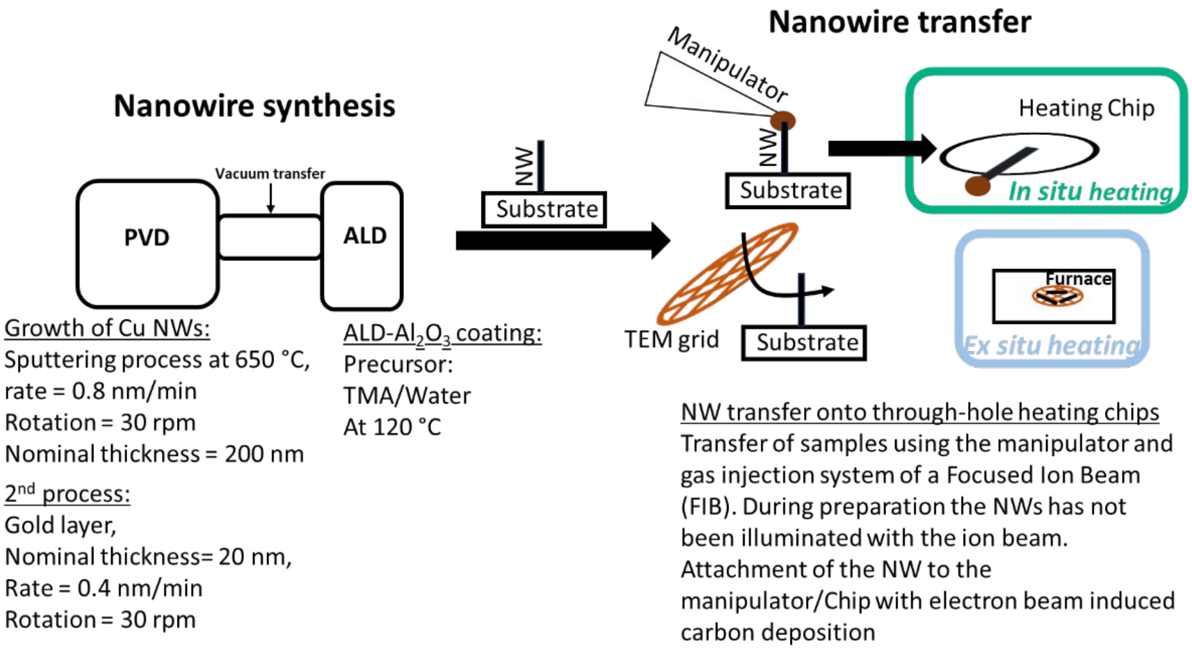

Supporting Figure 1. Schematic illustration of the sample preparation process.

Supporting Information 2

Example of a NW heated under beam-off conditions (no acquisition of EDX maps/STEM images during the heating procedure). The NW shows the same behavior has a NW under *in situ* conditions.

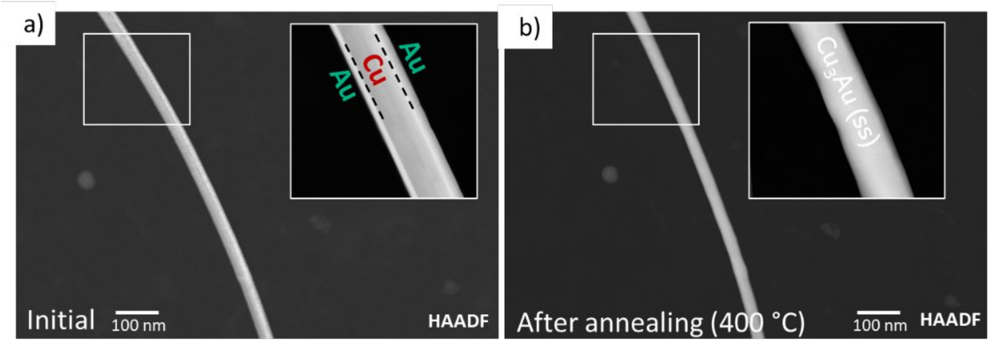

Supporting Figure 3. Heating experiment under beam-off conditions. a) initial NW with core-shell structure. During heating the NW has not been exposed to the electron beam. b) HAADF image of the same NW after heating at 400 °C for 1h.

Supporting Information 3

Example of *ex situ* annealed core-shell (Cu-Au) NWs using a furnace at 350 °C for 1h. After heating, the NW shows a homogeneous composition of Cu and Au. In HR STEM, we see the Cu_3Au (ordered) structure.

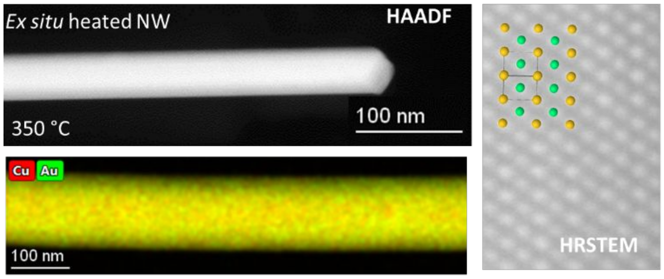

Supporting Figure 2. HAADF image of a NW after annealing in the furnace. The EDX map shows a homogenous composition of Au and Cu. HRSTEM reveals the ordered $L1_2$ structure of Cu_3Au (ordered) phase.

Supporting Information 4

Figure 1.1 provides an overview of the considerations taken into account to calculate diffusion coefficients based on our experimental data. Figure 1a illustrates schematically the projection of a core-shell nanostructure (Cu core, Au shell) in TEM.

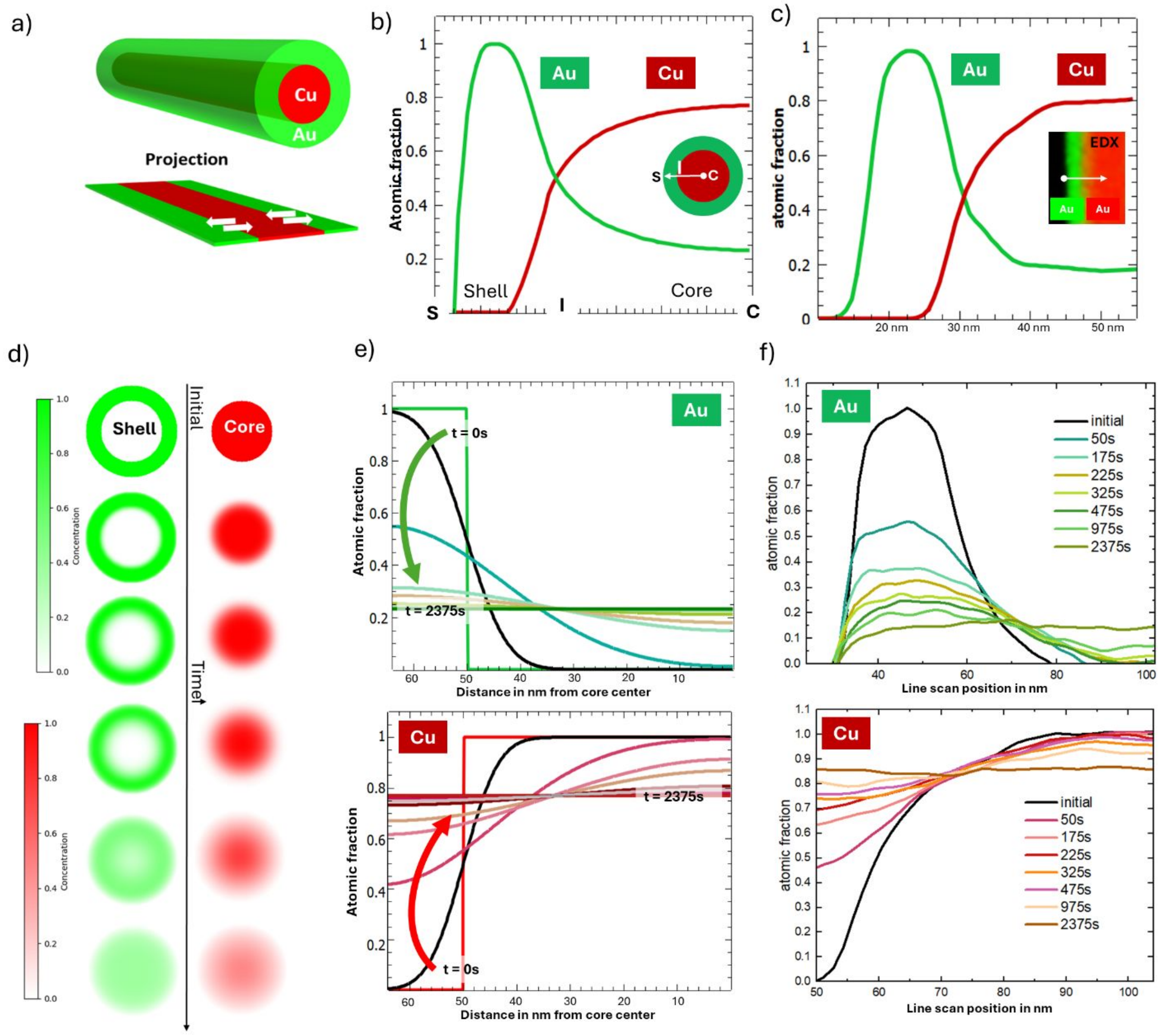

Supporting Figure 4.1. a) Schematic illustration of the Cu-Au sample system. b) Calculated projected atomic fraction of a core-shell structure along the beam direction. b) Simulated diffusion of a core-shell structure by using Fick's laws of diffusion. c)Experimental EDX profile of core-shell structure. Inset: EDX map and indicated position of the line scan d) Calculated concentration profiles (of Au and Cu) per time during interdiffusion (line scan across core-shell structure). Dimensions and diffusion coefficients are taken from the experiment. e) Experimental EDX data obtained during *in situ* heating in TEM.

Corresponding to this, figure 1b shows the calculated projected atomic fraction of such a structure. The calculated profile was convolved with a gaussian function that simulates the finite resolution of the electron beam. Figure 1c shows an experimental EDX line profile across a core-shell NW. To calculate the diffusion coefficient, we take the projected profile into account and the relative changes in composition during heating are considered. Figure 1d is a simulated diffusion process of a core-shell structure according to Ficks law of diffusion. The interdiffusion of the core and the shell is radially homogeneous. Figure 1e shows calculated concentration profiles per time during interdiffusion (line scan across core-shell structure) based on the dimensions and diffusion coefficient obtained in the experiment. Figure 1f shows the corresponding experimental EDX profiles. The calculated concentration profiles and the experimental data show the same progression with evolving time. The interdiffusion coefficients are calculated by using the equation developed by Wagner for a binary diffusion couple[6]:

$$\tilde{D}(Y^*) = \frac{V_m}{2t}\left(\frac{dx}{dY}\right)^* \left[(1 - Y^*)\int_{x^{-\infty}}^{x^*} \frac{Y}{V_m} dx + Y^* \int_{x^*}^{x^{+\infty}} \frac{1 - Y}{V_m} dx\right]$$

* Represents the selected point of interest

Normalizing composition variable Y:

$$Y = \frac{N_i - N_i^-}{N_i^+ - N_i^-}$$

N_i: atom fraction of the component i

N_i^- / N_i^+ : composition on the left/right side

V_m: molar volume

x: position parameter

t: annealing time

R. Ravi and A. Paul demonstrated the general applicability of the equation to binary diffusion couples[7]. In our work we follow the approach to calculate diffusion coefficients based on composition profiles. The calculation takes the composition on the left and right hand side of the diffusion couple into account. The molar volume V_m is calculated based on the change in lattice parameter a with composition:

$a = 0.408\ N_{Au} + 0.36(1\text{-}N_{Au})$

$V_m = N_A*(a^3/4)$ N_A: Avogadro's number

Figure 2 shows the calculation procedure based on our experimental data (example: Au line profile after annealing at 400 °C) for $N_{Au} = 0.2$. Figure 2a shows a plot of Y versus x, where we take the slope at the point of interest ($N_{Au} = Y$). Figure 2b and 2c show the integrals (shaded areas) used to calculate diffusion coefficients. Based on the extracted values, we calculated the interdiffusion coefficient.

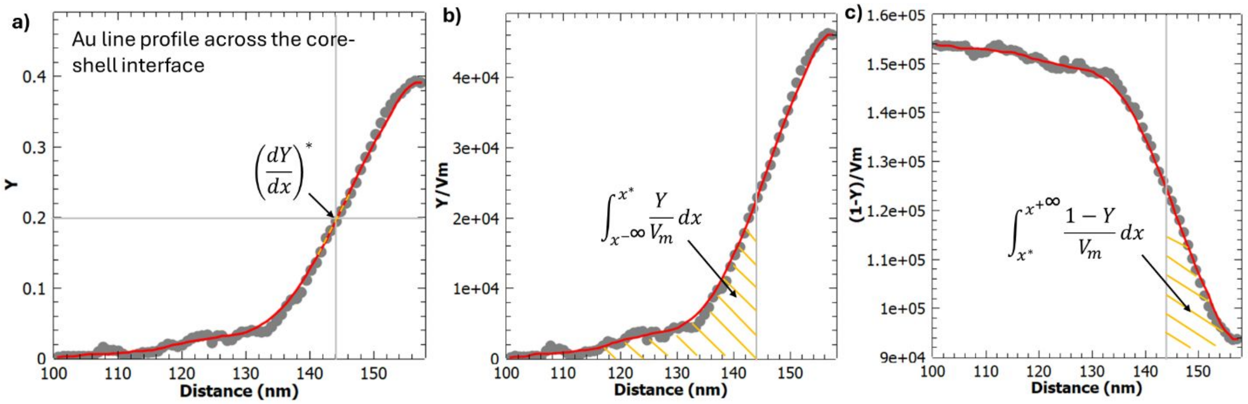

Supporting Figure 4.2. EDX line profile of Au after annealing at 400 °C. a) Y plotted against x. b) Y/Vm is plotted against x. c) (1-Y)/Vm is plotted against x.

In general, the calculation of interdiffusion coefficients are related to the diffusion fluxes following Fick's laws[8]. The Matano-Boltzmann method is based on simplifying the partial differential equations of Ficks second law [9] into ordinary differential equations based on the Boltzmann parameter[10]. However, this method relies on a precise determination of the Matano plane. Based on that, Wagner has proposed a method to bypass the Matano plane location in the analysis of binary interdiffusion. The Wagner equation represents an analytical approach based on algebraic equations without the need to locate the Matano plane graphically. The details of this approach can be found in text books[11,12]. It considers the actual variation of molar volume with composition and is widely used to determine interdiffusion coefficients in binary systems [13] [14] [7]**.** A. Gusak and N. Storozhuk demonstrated the applicability of coefficients based on Wagner's equation within atomistic-scale models[15].

Supporting Information 5

CTEM image corresponding to the diffraction pattern shown the main manuscript in figure 3c.

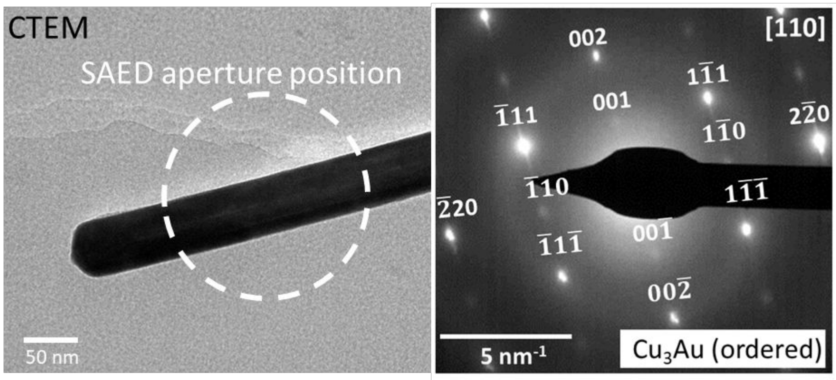

Supporting Figure 5. CTEM image of the Cu_3Au NW (left) and the corresponding diffraction pattern (right). The NW is oriented in [110] zone axis. The aperture position is indicated with the white dashed circle.

Supporting Information 7

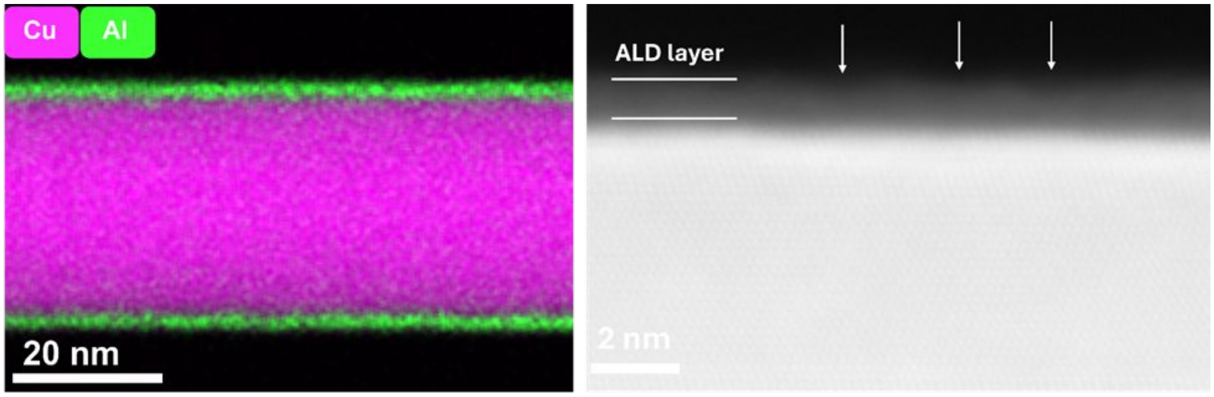

Supporting Figure 7. EDX map of a 1 nm Al_2O_3 shell on a copper NW as well as a high resolution STEM image of such a layer. While the shell looks largely homogenous in the EDX map, the high resolution image reveals several inhomogeneities in the film (marked with white arrows).

References used in the Supporting Information

(1) Vogl, L. M.; Schweizer, P.; Pethö, L.; Sharma, A.; Michler, J.; Utke, I. From Metal Nanowires to Ultrathin Crystalline ALD Nanotubes: Process Development and Mechanism Revealed by in Situ TEM Heating Experiments. *Nanoscale* **2023**, *15* (21), 9477–9483. https://doi.org/10.1039/D3NR01185B.

(2) Vogl, L. M.; Schweizer, P.; Minor, A. M.; Michler, J.; Utke, I. Unraveling the Highly Plastic Behavior of ALD-Aluminum Oxide Encapsulations by Small-Scale Tensile Testing. *Advanced Engineering Materials* **2024**, *26* (8), 2302220.

(3) Schweizer, P.; Vogl, L. M.; Maeder, X.; Utke, I.; Michler, J. Optimizing Atomic Layer Deposition Processes with Nanowire-Assisted TEM Analysis. *Advanced Materials Interfaces* **2024**, *n/a* (n/a), 2301064. https://doi.org/10.1002/admi.202301064.

(4) Vogl, L. M.; Schweizer, P.; Richter, G.; Spiecker, E. Effect of Size and Shape on the Elastic Modulus of Metal Nanowires. *MRS Advances* **2021**, *6*, 665–673.

(5) Vogl, L. M.; Schweizer, P.; Denninger, P.; Richter, G.; Spiecker, E. Sensing Capabilities of Single Nanowires Studied with Correlative In Situ Light and Electron Microscopy. *ACS Nano* **2022**, *16* (11), 18110–18118. https://doi.org/10.1021/acsnano.2c04848.

(6) Wagner, C. The Evaluation of Data Obtained with Diffusion Couples of Binary Single-Phase and Multiphase Systems. *Acta Metallurgica* **1969**, *17* (2), 99–107. https://doi.org/10.1016/0001-6160(69)90131-X.

(7) Ravi, R.; Paul, A. Diffusion Mechanism in the Gold-Copper System. *Journal of Materials Science: Materials in Electronics* **2012**, *23* (12), 2152–2156. https://doi.org/10.1007/s10854-012-0729-2.

(8) Baheti, V. A.; Paul, A. Development of Different Methods and Their Efficiencies for the Estimation of Diffusion Coefficients Following the Diffusion Couple Technique. *Acta Materialia* **2018**, *156*, 420–431. https://doi.org/10.1016/j.actamat.2018.04.051.

(9) Matano, C. On the Relation between the Diffusion-Coefficients and Concentrations of Solid Metals. *Japan. J. Phys.* **1933**, *8*, 109.

(10) Boltzmann, L. Zur Integration Der Diffusionsgleichung Bei Variabeln Diffusionscoefficienten. *Annalen der Physik* **1894**, *289* (13), 959–964.

(11) Paul, A. Estimation of Diffusion Coefficients in Binary and Pseudo-Binary Bulk Diffusion Couples. In *Handbook of Solid State Diffusion, Volume 1*; Elsevier, 2017; pp 79–201.

(12) Paul, A.; Laurila, T.; Vuorinen, V.; Divinski, S. V. Thermodynamics, Diffusion and the Kirkendall Effect in Solids. **2014**.

(13) Wang, Y.; Peng, X.; Huang, J.; Ye, Z.; Yang, J.; Chen, S. Studies of Cu-Sn Interdiffusion Coefficients in Cu3Sn and Cu6Sn5 Based on the Growth Kinetics. *Scripta Materialia* **2021**, *204*, 114138. https://doi.org/10.1016/j.scriptamat.2021.114138.

(14) Paul, A.; Ghosh, C.; Boettinger, W. Diffusion Parameters and Growth Mechanism of Phases in the Cu-Sn System. *Metallurgical and materials transactions A* **2011**, *42*, 952–963.

(15) Gusak, A. M.; Storozhuk, N. Two Remarks on Wagner Integrated Diffusion Coefficient. *Metallophysics and Advanced Technologies* **2019**, *41* (5), 583–593.
